# Long-term respiratory consequences of COVID-19 related pneumonia: a cohort study

**DOI:** 10.1186/s12890-023-02627-w

**Published:** 2023-11-11

**Authors:** Saioa Eizaguirre, Gladis Sabater, Sònia Belda, Juan Carlos Calderón, Victor Pineda, Marc Comas-Cufí, Marc Bonnin, Ramon Orriols

**Affiliations:** 1 Department of Respiratory, Dr. Josep, Trueta University Hospital of Girona, and Santa Caterina Hospital of Salt, Girona Biomedical Research Institute (IDIBGI), Girona, Catalonia Spain; 2 Department of Radiology, Dr. Josep, Trueta University Hospital of Girona, and Santa Caterina Hospital of Salt, Girona Biomedical Research Institute (IDIBGI), Girona, Catalonia Spain; 3 Department of Medical Sciences, Faculty of Medicine, University of Girona, Girona, Catalonia Spain; 4 Department of Computer Science, Mathematics and Statistics, University of Girona, Girona, Catalonia Spain; 5 Biomedical Research Networking Centre On Respiratory Diseases (CIBERES), Madrid, Spain

**Keywords:** COVID-19, Coronavirus disease, Long-term sequelae, Pneumonia, Pulmonary fibrosis

## Abstract

**Background:**

Our aims were to describe respiratory sequelae up to 12 months after discharge in COVID-19 patients with severe pneumonia requiring non-invasive respiratory support therapies.

**Methods:**

This study was undertaken at University Hospital Doctor Josep Trueta (Girona, Spain) between March 2020 and June 2020. Three months after discharge, we evaluated their dyspnoea and performed Saint George’s respiratory questionnaire, pulmonary function tests, blood test, 6-min walking test, and a high-resolution CT (HRCT). At the six and 12-month follow-up, we repeated all tests except for pulmonary function, 6-min walking test, and HRCT, which were performed only if abnormal findings had been previously detected.

**Results:**

Out of the 94 patients recruited, 73% were male, the median age was 62.9 years old, and most were non-smokers (58%). When comparing data three and 12 months after discharge, the percentage of patients presenting dyspnoea ≥ 2 decreased (19% vs 7%), the quality-of-life total score improved (22.8% vs 18.9%; *p* = 0.019), there were less abnormal results in the pulmonary function tests (47% vs 23%), the 6-min walking test distance was enhanced (368.3 m vs 390.7 m, *p* = 0.020), ground glass opacities findings waned (51.6% vs 11.5%), and traction bronchiectasis increased (5.6% vs 15.9%). Only age showed significant differences between patients with and without pulmonary fibrotic-like changes.

**Conclusion:**

Most patients improved their clinical condition, pulmonary function, exercise capacity and quality of life one year after discharge. Nonetheless, pulmonary fibrotic-like changes were observed during the follow-ups.

**Supplementary Information:**

The online version contains supplementary material available at 10.1186/s12890-023-02627-w.

## Background

Coronavirus disease 2019 (COVID-19) is caused by the severe acute respiratory syndrome coronavirus 2 (SARS-CoV-2). The high infectivity and virulence of the virus compelled the World Health Organization to declare COVID-19 a global pandemic in March 2020 [1]. As of March 2023, COVID-19 has caused more than 761 million confirmed cases and over than 6.8 million deaths have been reported worldwide [1]. During the first wave of the COVID-19 pandemic, about 10% to 20% of COVID-19 patients developed severe pneumonia and 20% to 25% required non-invasive respiratory support therapies (NIRT) [2]. A recent meta-analysis on one-year survivors showed that as much as 32.8% retained dyspnoea, 30.5% decreased DLCO, and around 32.6% showed radiological sequelae [3]. The early predominant radiological alteration on chest CT in recovered patients is ground-glass opacity (GGO), which could be reabsorbed and reversible [4]. However, the long-term evolution of these radiological findings requires monitoring as it could lead to fibrotic-like changes [3]. Therefore, an early diagnosis of potential reversible sequelae and identifying high-risk patients could be essential to apply a promptly treatment and prevent a possible evolution into irreversible damage.

The aims of this study were, first, to describe, one year of discharge, the respiratory consequences of COVID-19 in hospitalized patients who required NIRT. Second, to identify the incidence, risk factors, and impact of developing pulmonary fibrotic-like changes in those patients.

## Methods

### Study design

This observational, prospective, single-centre follow-up study was undertaken at the Respiratory Department of the University Hospital Doctor Josep Trueta, in Girona, Spain. We collected data from all patients with laboratory confirmed COVID-19 and a related severe pneumonia requiring NIRT between March 2020 and June 2020 who were discharged from our department.

### Patients

We included data from patients > 18 years old with SARS-CoV-2 severe pneumonia diagnosed by PCR and who had required NIRT (high-flow nasal cannula, continuous positive airway pressure, or non-invasive ventilation). We excluded data from patients who declined to participate.

### Procedures

The characteristics and comorbidities of patients were collected from their medical records. Three months after discharge, we evaluated dyspnoea according to the modified Medical Research Council scale [5]. We also performed a Saint George’s respiratory questionnaire (SGRQ) [6] a blood test (including lymphocyte count, LDH, ferritin and C-reactive protein), pulmonary function tests, a 6-min walking test (6MWT), and a chest high-resolution computed tomography (HRCT). The pulmonary function tests, undertaken in the Lung Function Laboratory of our Hospital using the Master Screen PFT (Jagger, Germany), and the 6-min walking test were performed according to the ATS/ERS guidelines [7]. We performed the chest HRCT in supine position during end-inspiration with multislice CT scanner. Images were reconstructed at 1 mm slice thickness, with 1 mm increment, 512 mm × 512 mm. CT features were reviewed, separately, by a pulmonologist and a radiologist with clinical experience in chest imaging. The main CT patterns were described, in line with the terms defined by the Fleischner Society and peer-reviewed literature on viral pneumonia [8, 9]. CT images were examined for the presence of parenchimal pattern (GGO or consolidation), reticular pattern (fine subpleural reticulation, coarse linear, or curvilinear opacity), and fibrotic pattern (traction bronchiectasis or bronchiolectasis, architectural distortion, or honeycombing) [9].

We reevaluated dyspnoea and performed again the SGRQ and the blood test six and 12 months after discharge. We performed pulmonary function tests, the 6MWT, and the HRCT only in those patients who had shown significant abnormal findings in that specific test at the previous evaluation. We considered abnormal findings a forced vital capacity (FVC) or diffusing capacity for carbon monoxide (DLCO) < 80% for pulmonary function tests and a distance walked below that of reference according to Enright criteria [10] for the 6-min walking test. Any abnormal finding in the chest HRCT was also considered relevant and was revaluated in the next evaluation.

At any of the follow-ups, all patients presenting significant GGO were treated with prednisone. We quantified the GGO in CT images with a modified, previously published method [9]. Briefly, each lung was divided into three zones: upper (above the carina), middle, and lower (below the inferior pulmonary vein). We considered a significant GGO involvement if more than one zone out of the six defined for both lungs showed signs of GGO. The initial prednisone regimen was 30 mg/day for 15 days and was gradually tapered between one to two months to 5 mg/day, a dose that was to be maintained until the next follow-up. Then, if the clinician considered that the CT results had improved resulting in a non-significant GGO, the treatment was interrupted.

### Statistical analysis

At baseline, we described categorical variables as absolute numbers and percentages and quantitative variables as mean and standard deviation (SD). To compare categorical variables, we used the Fisher's Exact test, to compare numerical variables, we used the Welch's t-test. We used a multivariate logistic model to estimate the adjusted odd-ratios (OR) of developing fibrotic-like changes. The candidate variables for adjustment were all available basal variables. We built the final model by using the set of variables which maximized the Bayesian Information Criterion. During the follow-up, to take into account repeated measures and missing values, we compared periods by fitting linear mixed-effect models using all patients available at baseline. For categorical variables, we present estimations in percentage together with their standard error. We used the likelihood ratio test to assess differences between periods. For quantitative variables, we present estimations with the standard error in parentheses. To compare periods, we considered the F-test based on the Kenward-Roger approach. We carried out all analysis using R (version 4.0.3).

## Results

**Fig. 1:**
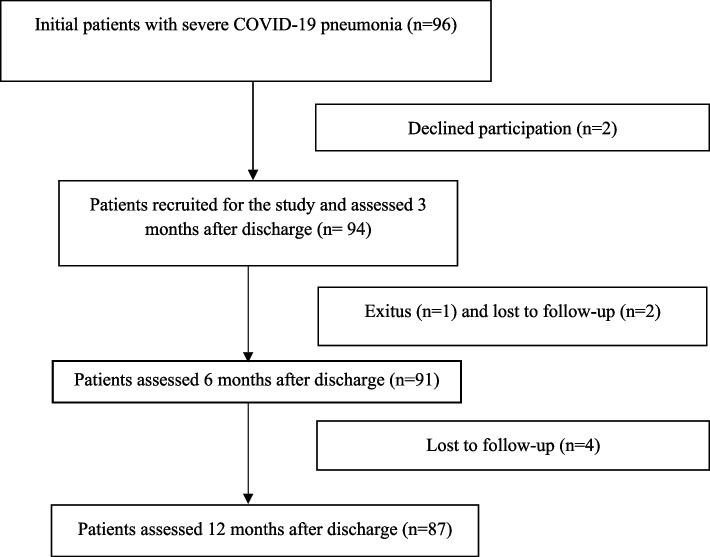
Flow chart describing patient recruitment

A total of 96 patients were initially considered, but two declined participation, hence, 94 patients were recruited for the study. One of them died and six dropped out during the follow-up, consequently, 87 patients completed the 12-month follow-up (Fig. 1). Our cohort was predominantly male (73%), with a mean age of 62.9 years (SD: 13.2) and a mean body mass index of 29.81 kg/m^2^ (SD: 5.23). The majority were non-smokers (57%). The most common comorbidities were hypertension (48%) and dyslipidaemia (34%), and the least frequent were cardiovascular diseases (21%). The median length of stay was 22 days (range: 3–87), and 46 patients (49%) were admitted to the ICU (Table 1).

**Table 1:** Baseline characteristics of study participants. Figures are absolute numbers (and %) unless otherwise stated

	**Total** (*n* = 94)
**Sex**	
Male	69 (73)
Female	25 (27)
**Age (years), *mean* (SD)**	62.9 (13.2)
**BMI (kg/m**^**2**^**), *mean* (SD)**	29.8 (5.2)
**Smoking history**	
Current	4 (4)
Former	36 (38)
Never	54 (58)
**Comorbidities**	
Hypertension	45 (48)
Dyslipidaemia	32 (34)
Diabetes mellitus	23 (24)
Respiratory diseases	23 (24)
Cardiovascular diseases	20 (21)
**Length of hospital stay (days), *median* [range]**	22 [3–87]
**ICU admission**	46 (49)

*BMI* Body mass index, *ICU* Intensive care unit, *SD* Standard deviation

Three months after discharge, 19% of patients presented dyspnoea ≥ 2, whereas at the 12-month follow-up that number decreased to 7% (*p* = 0*.*016) (Table 2). The results of the blood tests did not show any statistically significant differences between the three visits. Except for the symptoms score, quality-of-life parameters improved between the 3- and 6-month visits. However, all parameters improved when comparing the 3- and 12-month visits, but only impact score (15*.*6% vs 11*.*1%, *p* = 0*.*009) and total score (22*.*8% vs 18*.*9%, *p* = 0*.*019) differences were statistically significant (Table 2). The results of pulmonary function tests showed that most parameters improved between the 3- and 12-month follow-ups, although not always significantly (Table 3). The mean total lung capacity (97.2 vs 92.9, *p* = 0.063) and residual volume (100.6 vs 87.9, *p* = 0.001) worsened in this period. Notably, at the 3-month follow-up, 45 (47.8%) patients presented a FVC or DLCO < 80%, whereas at the 12-month visit 22 (23.4%) patients were below that threshold. On the other hand, 14% of patients presented an abnormal exercise capacity in the 6MWT 12 months after discharge. However, the mean distance walked increased significantly at the 6- (414*.*4 m, *p* = 0*.*002) and 12-month follow-up (390*.*7 m, *p* = 0*.*020) with respect to the 3-month visit (368*.*3 m). Oxygen saturation parameters remained stable throughout the follow-up (Table 3).

**Table 2:** Dyspnoea, blood test, and quality of life of our cohort at 3, 6, 12-month follow-up

	**3 months AD**	**6 months AD**	***p*-value**^1^	**12 months AD**	***p*-value**^2^
**Dyspnoea *%* (SE)**^3,4^			0.099		0.012*
0–1	79.9 (4.3)	88.9 (3.3)		92.9 (2.6)	
2–3	20.1 (4.2)	11.2 (3.3)		7.1 (2.6)	
**Blood test, *mean* (SE)**^4^					
Lymphocytes, K/µL	2.28 (0.1)	2.26 (0.1)	0.861	2.31 (0.1)	0.708
LDH, U/I	195.13 (5.5)	202.32 (5.5)	0.065	191.77 (6.2)	0.477
Ferritin, ng/mL	120.26 (10.6)	126.23 (10.8)	0.411	118.60 (11.9)	0.848
C-reactive protein, mg/dl	0.42 (0.1)	0.34 (0.1)	0.631	0.62 (0.2)	0.305
**Quality of life, *%* (SE)**^4,5^					
Symptoms score, %	22.3 (1.8)	26.7 (1.8)	0.026*	19.8 (1.9)	0.220
Activity score, %	35.7 (2.8)	27.8 (2.9)	0.002*	32.4 (3.0)	0.223
Impact score, %	15.6 (1.8)	13.5 (1.8)	0.194	11.1 (1.9)	0.009*
Total score, %	22.8 (1.9)	20.0 (1.9)	0.073	18.9 (2.0)	0.019*

*AD* After discharge, *LDH* Lactate dehydrogenase, *SE* Standard error

^1^*p*-value calculated with the paired sample t-test between 3 and 6 months AD

^2^*p*-value calculated with the paired sample t-test between 3 and 12 months AD

^3^Measured with the modified Medical Research Council dyspnea scale

^4^Estimates from the linear mixed-effect model considering all 94 patients

^5^According to Saint George’s respiratory questionnaire

^*^Statistically significant values (*p* < 0.05)

**Table 3:** Pulmonary function, exercise function, and HRCT findings of our cohort at 3, 6, 12-month follow-up

	**3 months AD**	**6 months AD**	***p*-value**^1^	**12 months AD**	***p*-value**^2^
**Pulmonary function, *%* (SE)**^3^					
FVC predicted	94.9 (1.8)	97.1 (2.4)	0.271	101.2 (2.3)	0.002*
FEV_1_ predicted	95.2 (2.2)	98.4 (2.8)	0.153	100.0 (2.7)	0.026*
TLC predicted	97.2 (1.7)	97.6 (2.5)	0.875	92.9 (2.4)	0.063
RV predicted	100.6 (2.5)	102.6 (3.7)	0.593	87.9 (3.6)	0.001*
DLCO predicted	75.9 (1.8)	81.6 (2.6)	0.021*	80.6 (2.5)	0.051
KCO predicted	98.5(1.7)	106.3 (2.6)	0.005*	96.2 (2.5)	0.390
**Exercise function, *mean* (SE)**^3,4^					
Distance, meters	368.3 (8.8)	414.4 (15.7)	0.002*	390.7 (11.2)	0.020*
Basal oxygen saturation, %	97.9 (0.1)	97.5 (0.2)	0.148	97.5 (0.1)	0.008*
Mean oxygen saturation. %	96.3 (0.2)	95.7 (0.5)	0.201	95.8 (0.3)	0.067
Minimal oxygen saturation. %	95.4 (0.2)	94.0 (0.6)	0.039*	94.3 (0.4)	0.010*
**HRCT findings, *%* (SE)**^3^					
Non-pathological CT scan	21.5 (5.0)	12.1 (6.4)	0.305	29.9 (8.6)	0.390
Pathological CT scan	78.5 (5.0)	87.9 (6.4)	0.305	70.1 (8.6)	0.390
*Parenchimal pattern*	53.9 (6.7)	40.3 (9.7)	0.256	9.9 (0.49)	< 0.001*
GGO	51.6 (6.5)	42.7 (9.5)	0.440	11.5 (4.0)	< 0.001*
Consolidation	1.1 (1.1)	ND	1.000	ND	1.000
*Reticular pattern*	39.0 (7.4)	53.3 (12.3)	0.319	31.9 (9.5)	0.556
Fine subpleural reticular	37.0 (7.6)	51.8 (13.1)	0.327	25.4 (8.8)	0.325
Coarse linear or curvilinear opacities	14.6 (5.0)	15.3 (7.2)	0.932	2.9 (1.7)	0.010*
*Fibrosis pattern*	19.5 (5.6)	26.7 (10.1)	0.495	27.3 (9.5)	0.438
Traction bronchiectasis	5.6 (2.4)	11.5 (5.6)	0.230	15.9 (6.6)	0.062
Honeycomb	ND	ND	1.000	1.8 (1.8)	1.000
Architectural distortion	21.3 (5.6)	16.8 (6.8)	0.609	4.9 (2.6)	0.008*

*AD* After discharge, *DLCO* Diffusing capacity for carbon monoxide, *FEV*_*1*_ Forced expiratory volume during first second, *FVC* Forced vital capacity, *GGO* Ground glass opacity, *HRCT* High resolution computed tomography, *KCO* Carbon monoxide transfer coefficient, *ND* Not detected, *RV* Residual volume, *SE* Standard error, *TLC* Total lung capacity

^1^*p*-value calculated with the paired sample t-test between 3 and 6 months AD

^2^*p*-value calculated with the paired sample t-test between 3 and 12 months AD

^3^Estimates from the linear mixed-effect model considering all 94 patients

^4^According to 6-min walking test

^*^Statistically significant values (*p* < 0.05)

**Fig. 2:**
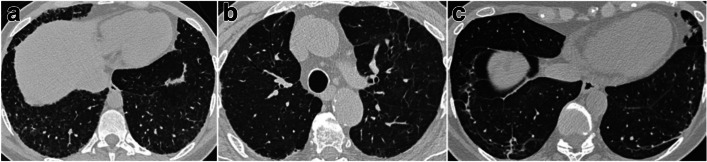
HRCT images showing diferent fibrosis patterns. **a** Honeycomb. **b** Traction bronchiectasis. **c** Architectural distorsions

The HRCT performed three months after discharge showed pathological signs in 78.5% of our cohort (Table 3). The most common feature found was GGO (51.6%), and the least common—apart from consolidation and honeycomb that were not detected at some visits—was traction bronchiectasis (5.6%) (Fig. 2). The presence of some features, such as GGO (*p* < 0.001), coarse linear or curvilinear opacities (*p* = 0.010), and architectural distortion (*p* = 0.008), showed a steady reduction between the 3- and 12-month visits, while the presence of fine subpleural reticular increased at the 6-month follow-up and decreased at 12-month follow-up, although not significantly. The percentage of patients with traction bronchiectasis (5.6% vs 11.5% vs 15.9%, *p* = 0.062) increased at the two visits following the 3-month initial evaluation (Table 3).

In the HRCT performed 12 months after discharge, 24 (28%) patients presented signs of fibrotic-like changes while 63 (72%) did not (Supplementary table 1). When analysing separately the baseline characteristics of patients with and without fibrotic-like changes, the only statistically significant differences were found in age (univariate analysis: OR: 1.103; 95% confidence interval [CI]: 1.030 – 1.182; multivariate analysis: OR: 1.943; 95% CI: 1.274 – 3.171) (Table 4).

**Table 4:** Univariate and multivariate analyses of the risk factors of developing pulmonary fibrotic-like changes in HRCT

	**Univariate analysis, OR (95% CI)**	**Multivariate analysis, OR (95% CI)**
Sex		
Male	1.098 (0.888 – 1.357)	
Female	0.911 (0.737 – 1.126)	
Age (10 years)	1.103 (1.030 – 1.182)	1.943 (1.274 – 3.171)
BMI (1 kg/m^2^)	0.991 (0.973 – 1.009)	
Smoking history		
Current	0.749 (0.478 – 1.174)	
Former	1.097 (0.903 – 1.333)	
Never		
Comorbidities		
Hypertension	0.930 (0.769 – 1.124)	
Dyslipidemia	1.038 (0.850 – 1.267)	
Diabetes mellitus	0.984 (0.782 – 1.238)	
Respiratory diseases	1.058 (0.851 – 1.316)	
Cardiovascular diseases	1.153 (0.914 – 1.455)	
Length of hospital stay (1 day)	1.000 (0.995 – 1.006)	
ICU admission	0.961 (0.795 – 1.162)	

*HRCT* High-resolution computed tomography, *OR* Odd ratios, *CI* Confidence interval

## Discussion

In this study, we analysed the respiratory consequences of COVID-19 in patients hospitalized because of pneumonia who required NIRT. In our cohort, dyspnoea, quality of life, pulmonary function, and exercise capacity improved between the 3- and 12-month visits. However, HRCT scans showed that the percentage of patients with fibrotic-like changes increased six and 12 months after discharge with respect to the initial evaluation at the 3-month follow-up. The high percentage of fibrotic-like changes observed in our study, compared to a previous meta-analysis [3] could be explained by the fact that all patients included suffered a respiratory failure requiring NIRT and that severity has been clearly associated with fibrotic-like changes in COVID-19 patients.In addition, the mean age of our patients, previously identified as a risk factor [1], was higher than that of the studies included in that meta-analysis [3]. In our study, the number of patients with dyspnoea MRC ≥ 2 steadily decreased at all follow-ups and quality of life improved progressively. These facts were concordant with overall improving pulmonary function and exercise capacity during the study period. However, lung function and exercise capacity abnormalities, particularly a reduced DLCO, were found after hospital discharge. This abnormal functional test has already been reported as the most frequent and persistent change after COVID-19 pneumonia [3], which has been attributed to interstitial abnormalities, pulmonary vascular abnormalities [11] and, even, to air trapping due to distal airways abnormalities [12]. In this sense, the decrease in residual volume 12 months after discharge with respect to the 3-month visit value in our study suggests an improvement in distal airways obstruction. This, in turn, could have contributed to improving dyspnoea, exercise capacity, and quality of life.

A significant and progressive reduction in the percentage of patients with GGO was observed in our study. Besides, the percentage of patients with reticular pattern in our cohort increased at the 6-month follow but decreased at the 12-month visit with respect to the 3-month initial evaluation. This could indicate an initial absorption from alveolar pathologic substrate of GGO to the interstitial area at six months and a partial resolution at 12 months. Architectural distortion, a feature that has been considered related to fibrosis [9], improved significantly in our patients 12 months after discharge, which suggests that this change after suffering severe COVID-19 pneumonia could also be reversible in some patients. On the contrary, traction bronchiectasis, an accepted sign of irreversible lung fibrosis, showed an increasing trend. These changes were consistent with a radiological study [13], reporting that fibrotic-like changes in patients after COVID-19 pneumonia evolved from GGO, and with another pathological study [14] that found patchy collagenous scars as a remaining sequel of organizing pneumonia of COVID-19 pneumonia. Moreover, the disparity between the development of traction bronchiectasis and the clinical-functional improvement over 12 months in our study, in addition to a lack of differences in dyspnea and pulmonary function at 12-month follow-up between patients with or without fibrotic-lung changes, suggest that the observed fibrotic radiological signs are non-active scars rather than a feature of a classical interstitial lung disease or progressive pulmonary fibrosis [15]. Only age was found to be a risk factor for developing fibrotic-like changes. This finding is in line with that of other short-term follow-up studies where age was identified as a potential predictor of these abnormalities [13, 16, 17].

Following an internal multidisciplinary protocol, corticosteroids were initiated in patients presenting significant GGO. Although GGO and organizing pneumonia—the most frequent radiological and pathological abnormalities in post-COVID pneumonia—have been reported to improve with corticosteroids [18–20], the methodology (design) of our study did not allow us to infer the effect of this therapy in our patients. Other clinical trials are needed to prove or disprove the efficacy of this treatment.

Our findings must also be interpreted in light of the study’s limitations. First, our cohort was relatively small and was limited to patients discharged from hospital with a certain severity; second, we analyzed associations without adjusting for multiple comparisons, which can increase the risk of type I error, third, this was a single-centre study; and, forth, we did not have radiological and functional baseline data. In addition, although, the proportion of patients with comorbidities was considered in our analysis, these were self-reported by patients and might have resulted in underestimations.

## Conclusion

The dyspnoea, quality of life, pulmonary function, and exercise capacity of our COVID-19 patients hospitalized because of severe pneumonia and who required NIRT improved one year after discharge. On the contrary, fibrotic-like changes were observed by HRCT during the follow-ups. Besides, our study identified age as the sole risk factor of developing that damage. Our results add important information about the long-term consequences of patients requiring NIRT, a substantial part of COVID-19 patients with severe pneumonia. Further studies are necessary to better determine the long-term sequelae of COVID-19 related pneumonia and to put forward strategies to prevent them.

### Supplementary Information

**Additional file 1: Supplementary table 1.** Baseline characteristics of patients stratified by the presence of fibrotic-changes in HRCT 3 months after discharge.

**Additional file 2: Supplementary table 2.** Number of measurements available in each visit (or investigations performed at 3, 6, and 12 month-follow-up).

## Data Availability

The data that support the findings of this study are available from the corresponding author (RO) upon reasonable request and with permission of Dr. Josep Trueta University Hospital of Girona.

## Abbreviations

GGO: Ground-glass opacity
HRCT: High-resolution computed tomography
ICU: Intensive care unit
MRC: Medical Research Council scale
NIRT: Non-invasive respiratory support therapies
PFT: Pulmonary function test
SD: Standard deviation
SGRQ: Saint George’s respiratory questionnaire
6MWT: 6-min walking test
