# Supplementary material for: Long-term respiratory consequences of COVID-19 related pneumonia: a cohort study
Source: BMC Pulm Med. 2023 Nov 11;23:439. doi: 10.1186/s12890-023-02627-w (PMC10638724; doi:10.1186/s12890-023-02627-w)
Supplement: Supplementary file 1 — Additional file 1: Supplementary table 1. Baseline characteristics of patients stratified by the presence of fibrotic-changes in HRCT 3 months after discharge. [file 12890_2023_2627_MOESM1_ESM.docx]

| **Supplementary table 1. Baseline characteristics of patients stratified by the presence of fibrotic-changes in HRCT 3 months after discharge.** | | | |
| --- | --- | --- | --- |
|  | **PF patients**  (n=24) | **NPF patients**  (n=63) | **p-value**^1^ |
| **Sex** |  |  | 0.636 |
| Male | 19 (79) | 44 (70) |  |
| Female | 5 (21) | 19 (30) |  |
| **Age (years), *mean* (SD)** | 69.3 (9.7) | 60.7 (13.7) | 0.002* |
| **BMI (kg/m^2^), *mean* (SD)** | 29.2 (4.6) | 30.5 (5.4) | 0.496 |
| **Smoking history** |  |  | 0.551 |
| Current | 0 (0) | 4 (6) |  |
| Former | 11 (46) | 22 (35) |  |
| Never | 13 (54) | 37 (59) |  |
| **Comorbidities** |  |  |  |
| Hypertension | 10 (42) | 32 (511) | 0.639 |
| Dyslipidaemia | 9 (38) | 21 (33) | 0.869 |
| Diabetes mellitus | 5 (21) | 14 (22) | 0.838 |
| Respiratory diseases | 7 (29) | 15 (24) | 0.730 |
| Cardiovascular diseases | 7 (29) | 11 (17) | 0.421 |
| **C-reactive protein, mg/dl**  **(at admission)** | 22.3 (35.8) | 11.1 (8.7) | 0.160 |
| **Length of hospital stay (days), *median* [range]** | 23 [5-60] | 21 [3-87] | 0.956 |
| **ICU admission (days), *mean* (SD)** | 11 (45.8) | 32 (50.8) | 0.815 |
| **Dyspnoea (at 12-month follow-up), *n* (%)** | 0 (0.0) | 8 (12.7) | 0.103 |
| **Pulmonary function, %(se)** |  |  |  |
| FVC predicted | 103.4 (4.0) | 99.7 (2.9) | 0.457 |
| FEV1 predicted | 105.8 (4.9) | 96.9 (3.3) | 0.136 |
| TLC predicted | 90.6 (4.1) | 93.4 (3.0) | 0.583 |
| RV predicted | 78.3 (6.1) | 92.3 (4.4) | 0.062 |
| DLCO predicted | 84.3 (4.2) | 78.1 (3.1) | 0.239 |
| KCO predicted | 102.6 (4.2) | 92.6 (3.2) | 0.056 |
| BMI: Body mass index; HRCT: High-resolution computed tomography; ICU: Intensive care unit; NPF: Non-pulmonary fibrotic-changes; PF: Pulmonary fibrotic-changes; SD: Standard deviation. Figures are absolute numbers (and %) unless otherwise stated**.** ^1^p-values were calculated with Chi-square, Fisher’s exact test, Student’s t-test, or Mann-Whitney U test as appropriate.  *Statistically significant values (p <0.05). | | | |
