# Supplementary material for: Long-term respiratory consequences of COVID-19 related pneumonia: a cohort study
Source: BMC Pulm Med. 2023 Nov 11;23:439. doi: 10.1186/s12890-023-02627-w (PMC10638724; doi:10.1186/s12890-023-02627-w)
Supplement: Supplementary file 2 — Additional file 2: Supplementary table 2. Number of measurements available in each visit (or investigations performed at 3, 6, and 12 month-follow-up). [file 12890_2023_2627_MOESM2_ESM.docx]

| **Supplementary table 2. Number of measurements available in each visit (or Investigations performed at 3, 6, and 12 month-follow-up)** | | | |
| --- | --- | --- | --- |
| **Variable** | **1st visit** | **2nd visit** | **3rd visit** |
| **Dyspnea, % (se)** |  |  |  |
| 0-1 | 94 / 94 (100.0) | 89 / 94 (94.7) | 87 / 94 (92.6) |
| 2-3 | 94 / 94 (100.0) | 89 / 94 (94.7) | 87 / 94 (92.6) |
| **Blood test, mean (se)** |  |  |  |
| Lymphocytes, K/μL | 77 / 94 (81.9) | 73 / 94 (77.7) | 52 / 94 (55.3) |
| LDH, U/I | 76 / 94 (80.9) | 69 / 94 (73.4) | 39 / 94 (41.5) |
| Ferritin, ng/mL | 74 / 94 (78.7) | 68 / 94 (72.3) | 40 / 94 (42.6) |
| C-reactive protein, mg/dL | 76 / 94 (80.9) | 69 / 94 (73.4) | 47 / 94 (50.0) |
| **Quality of life, mean (se)** |  |  |  |
| Symptoms score, | 93 / 94 (98.9) | 85 / 94 (90.4) | 74 / 94 (78.7) |
| Activity score, | 93 / 94 (98.9) | 85 / 94 (90.4) | 74 / 94 (78.7) |
| Impact score, | 93 / 94 (98.9) | 85 / 94 (90.4) | 74 / 94 (78.7) |
| Total score, | 93 / 94 (98.9) | 85 / 94 (90.4) | 74 / 94 (78.7) |
| **Pulmonary function, (se)** |  |  |  |
| FVC predicted | 90 / 94 (95.7) | 29 / 56 (51.8) | 24 / 48 (50.0) |
| FEV₁ predicted | 90 / 94 (95.7) | 29 / 56 (51.8) | 24 / 48 (50.0) |
| TLC predicted | 86 / 94 (91.5) | 28 / 56 (50.0) | 24 / 48 (50.0) |
| RV predicted | 86 / 94 (91.5) | 28 / 56 (50.0) | 24 / 48 (50.0) |
| DLCO predicted | 89 / 94 (94.7) | 29 / 56 (51.8) | 24 / 48 (50.0) |
| KCO predicted | 89 / 94 (94.7) | 29 / 56 (51.8) | 24 / 48 (50.0) |
| **Exercise function, mean (se)** |  |  |  |
| Basal oxygen saturation, | 89 / 94 (94.7) | 5 / 42 (11.9) | 17 / 39 (43.6) |
| Mean oxygen saturation, | 89 / 94 (94.7) | 5 / 42 (11.9) | 16 / 39 (41.0) |
| Minimal oxygen saturation, | 89 / 94 (94.7) | 5 / 42 (11.9) | 16 / 39 (41.0) |
| TC meters | 89 / 94 (94.7) | 5 / 42 (11.9) | 16 / 39 (41.0) |

| **Supplementary table 2 (continued). Number of measurements available in each visit (or Investigations performed at 3, 6, and 12 month-follow-up)** | | | |
| --- | --- | --- | --- |
| **Variable** | **1st visit** | **2nd visit** | **3rd visit** |
| **Chest HRCT findings, (se**) |  |  |  |
| Pathological CT Scan | 94 / 94 (100.0) | 51 / 67 (76.1) | 52 / 63 (82.5) |
| Parenchimal pattern | 94 / 94 (100.0) | 51 / 67 (76.1) | 52 / 63 (82.5) |
| GGO | 94 / 94 (100.0) | 51 / 67 (76.1) | 52 / 63 (82.5) |
| Consolidation | 94 / 94 (100.0) | 51 / 67 (76.1) | 52 / 63 (82.5) |
| Reticular pattern= septal | 94 / 94 (100.0) | 51 / 67 (76.1) | 52 / 63 (82.5) |
| Fine subpleural reticular | 94 / 94 (100.0) | 51 / 67 (76.1) | 51 / 63 (81.0) |
| Coarse linear or curvilinear opacities | 94 / 94 (100.0) | 51 / 67 (76.1) | 52 / 63 (82.5) |
| Diffuse fibrosis | 94 / 94 (100.0) | 51 / 67 (76.1) | 52 / 63 (82.5) |
| Traction bronchiectasis | 94 / 94 (100.0) | 51 / 67 (76.1) | 52 / 63 (82.5) |
| Honeycomb | 94 / 94 (100.0) | 51 / 67 (76.1) | 52 / 63 (82.5) |
| Architectural distorsion | 94 / 94 (100.0) | 51 / 67 (76.1) | 50 / 63 (79.4) |
| Non-pathological CT Scan | 94 / 94 (100.0) | 51 / 67 (76.1) | 52 / 63 (82.5) |
